# Targeted Therapies in Breast Cancer: Implications for Advanced Oncology Practice

**DOI:** 10.6004/jadpro.2014.5.4.2

**Published:** 2014-07-01

**Authors:** Laura Bourdeanu, Thehan Luu

**Affiliations:** From The Sage Colleges, Troy, New York, and City of Hope National Medical Center, Duarte, California

## Abstract

The systemic therapeutic management of breast cancer has undergone significant transformation in the past decade. Without targeted therapies, conventional treatment with cytotoxic agents has reached the limit of its potential in terms of patient survival for most types of cancer. Enhanced understanding of the pathogenesis of tumor cell growth and metastasis has led to the identification of signaling growth pathways as targets for these directed therapies. Novel therapies targeted to HER2/*neu*, epidermal growth factor receptor (EGFR), vascular endothelial growth factor (VEGF), poly(ADP ribose) polymerase (PARP), mammalian target of rapamycin (mTOR), histone deacetylase (HDAC), the heat shock protein, and cyclin-dependent kinase (CDK) inhibitors have been developed and have demonstrated some efficacy in breast cancer. Recognition and management of the toxicities associated with targeted therapies is imperative. This review will describe the clinical development and utilization of targeted therapies currently in use or in clinical trials, with a focus on considerations for the oncology advanced practitioner.

During the past decade, the systemic therapeutic management of breast cancer has undergone a significant transformation. Without targeted therapies, conventional treatment with cytotoxic agents has maximized its potential in terms of patient survival for most types of cancer. Enhanced understanding of the pathogenesis of tumor cell growth and metastasis has led to the identification of signaling growth pathways as targets for these directed therapies.

##  Anti-HER2/*neu* Therapy

**Trastuzumab** 

The HER2/*neu* oncogene, a transmembrane tyrosine kinase receptor belonging to the epidermal growth factor receptor (EGFR) family, has been shown to be amplified in up to 30% of human breast cancer cell lines (Slamon et al., 1987). Identification of HER2/*neu* led to the development of trastuzumab (Herceptin), a humanized monoclonal antibody of the IgG1 type directed against the extracellular portion of human EGFR HER2/*neu*, and revolutionized the management of both early and advanced breast cancer. Pivotal phase II and III clinical trials of trastuzumab given in combination with chemotherapy to women with early-stage and metastatic breast cancer (MBC) have demonstrated that trastuzumab is associated with significantly longer overall survival (OS), longer time to tumor progression (TTP), and longer duration of response (Slamon et al., 2004; Romond et al., 2005; Piccart-Gebhart et al, 2005; Joensuu et al., 2006; Robert et al., 2006; Pierga et al., 2010; Marty et al., 2005; Inoue et al., 2010).

**Ado-Trastuzumab Emtansine** 

Ado-trastuzumab emtansine (Kadcyla) is an antibody-drug conjugate designed to combine the biological activity of trastuzumab with targeted delivery of a potent microtubule-disrupting agent, DM1 (a maytansine derivative), to HER2/*neu*-expressing cancer cells (Lewis Phillips et al., 2008). In a phase I study, ado-trastuzumab emtansine showed clinical activity in heavily pretreated patients with HER2/*neu*-overexpressing metastatic breast cancer (Krop et al., 2010).

The recommended dose for phase II trials was determined to be 3.6 mg/kg every 3 weeks. The phase II studies confirmed this strong activity in patients with HER2/*neu*-positive MBC whose disease progressed while receiving HER2/*neu*-directed therapy or who were previously treated with an anthracycline, a taxane, capecitabine, lapatinib (Tykerb), and trastuzumab, with overall response (OR) rates in the range of 23.9% to 39.5% (Burris 3rd et al., 2011).

The open-label phase III trial (EMILIA) comparing ado-trastuzumab emtansine vs. capecitabine and lapatinib in HER2/*neu*-positive locally advanced or metastatic breast cancer previously treated with trastuzumab and a taxane confirmed that ado-trastuzumab emtansine significantly improved progression-free survival (PFS, *p* < .001) and OS was not reached vs. 23.3 months (*p* = .005) compared with capecitabine and lapatinib (Blackwell et al., 2012).

The primary results from a phase III trial, called the TH3RESA trial, showed that ado-trastuzumab emtansine increased PFS in patients whose cancer was inoperable or had recurred or metastasized after several treatments including trastuzumab and lapatinib and were treated with ado-trastuzumab emtansine vs. physician’s choice of treatment (6.2 vs. 3.3 months, respectively, *p* < .0001). The interim analysis of OS showed a trend in favor of ado- trastuzumab emtansine, but it did not reach a level of statistically significant benefit (Wildiers et al., 2013).

**Pertuzumab** 

Pertuzumab (Perjeta) is a monoclonal antibody that binds to the dimerization domain of HER2/*neu* and prevents receptor dimerization, thus preventing HER2/*neu*-mediated intracellular signaling (Franklin et al., 2004). Data from a phase I trial demonstrated the dosage of pertuzumab to be > 5 mg/kg given every 3 weeks (Agus et al., 2005).

A phase II trial in patients with HER2/*neu*-negative disease suggested that pertuzumab had some activity as a single agent; however, the benefit was so limited that further investigation of single-agent pertuzumab in unselected patients with HER2/*neu*-negative disease was unwarranted (Gianni et al., 2010).

Another phase II trial assessed the efficacy and safety profile of pertuzumab in combination with trastuzumab in patients with HER2/*neu*-positive breast cancer whose disease had progressed during prior trastuzumab-based therapy. Patients received trastuzumab weekly (4 mg/kg loading dose, then 2 mg/kg every week) or every 3 weeks (8 mg/kg loading dose, then 6 mg/kg every 3 weeks) and pertuzumab every 3 weeks (840 mg loading dose, then 420 mg every 3 weeks). Treatment continued until disease progression or excessive toxicity. Overall, the combination of pertuzumab and trastuzumab was well tolerated, and adverse events were mild to moderate (Baselga et al., 2010).

A subsequent phase III trial (CLEOPATRA) assessed the activity of pertuzumab in patients with HER2/*neu*-positive adenocarcinoma of the breast with locally recurrent or metastatic disease. Patients were randomized (1:1) to receive docetaxel, trastuzumab, and pertuzumab or docetaxel, trastuzumab, and placebo. The median PFS increased significantly by 6.1 months in the pertuzumab group (hazard ratio [HR] for disease progression or death, 0.62; 95% confidence interval [CI] = 0.51–0.75; *p* < .001). The interim analysis of OS data showed a strong trend toward a survival benefit with pertuzumab/trastuzumab/docetaxel therapy, although it did not reach significance (Baselga & Swain, 2010).

**Side Effects** 

Although there are similarities in the side-effect profiles of all three of these drugs, there are some adverse events that are unique to each agent. The most common adverse reactions associated with trastuzumab include headache, diarrhea, nausea, chills, infection, congestive heart failure, insomnia, cough, and rash (Robert et al., 2006; Pierga et al., 2010; Marty et al., 2005; Inoue et al., 2010). The most common side effects associated with pertuzumab are diarrhea, alopecia, neutropenia, nausea, rash, and peripheral neuropathy. Finally, the most common side effects associated with ado-trastuzumab emtansine are thrombocytopenia, epistaxis, eye-tearing/conjunctivitis disorder, and elevated liver enzymes (Baselga et al., 2010; Baselga & Swain, 2010; Agus et al., 2005; Blackwell et al., 2012; Burris 3rd et al., 2011; Gianni et al., 2010; Krop et al., 2010).

One of the most concerning side effects of HER2/*neu* therapy is cardiac dysfunction or failure. Cardiac toxicity occurs in 7% to 28% of patients treated with trastuzumab alone or in combination with anthracycline-based chemotherapy, and in 1.2% of patients treated with pertuzumab in combination with chemotherapy (Agus et al., 2005; Baselga et al., 2010; Baselga & Swain 2010; Gianni et al., 2010; Inoue et al., 2010; Marty et al., 2005; Pierga et al., 2010; Robert et al., 2006; Slamon et al., 2001; Wardley et al., 2010). Anti-HER2/*neu* therapy–induced cardiac failure may be severe, and in some cases associated with death.

Other concerning grade 3 side effects of anti-HER2/*neu* therapy include neutropenia, leukopenia, thrombocytopenia, diarrhea, elevated liver enzymes, and palmar-plantar erythrodysesthesia (Agus et al., 2005; Baselga et al., 2010; Baselga & Swain, 2010; Gianni et al., 2010; Inoue et al., 2010; Marty et al., 2005; Pierga et al., 2010; Robert et al., 2006; Slamon et al., 2001; Wardley et al., 2010). These side effects have generally been observed when the therapy is used in combination with other antineoplastic agents. Other less common and grade < 3 side effects are listed in Table 1.

**Table 1:**
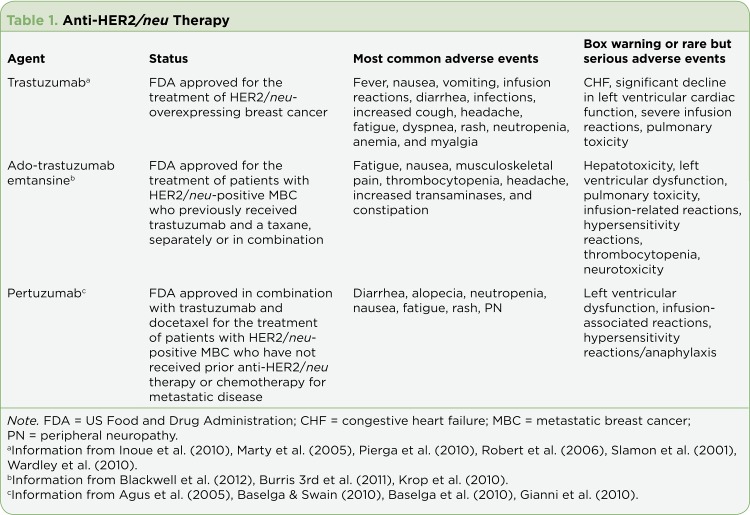
Anti-HER2/*neu* Therapy

## HER2 and EGFR Pathway Inhibitors

**Lapatinib** 

Lapatinib is a reversible dual EGFR/HER1 and HER2 tyrosine kinase inhibitor (TKI) that acts intracellularly, directly targeting the TK domains of HER1 and HER2 and inhibiting the receptor phosphorylation, leading to inhibition of downstream pathways that control cell proliferation and survival (Tevaarwerk & Kolesar, 2009). The combination of lapatinib and capecitabine showed clinical activity in a phase I study of patients with advanced solid tumors at a dose of 1,500 mg/day (Chu et al., 2007).

Several phase II trials examined the efficacy of lapatinib in HER2/*neu*-positive MBC patients who failed to respond to trastuzumab therapy. The OR rate was 4% to 8%, whereas 15% to 46% of patients had stable disease and 13% to 22% remained progression-free at 16 weeks after treatment with lapatinib (Burstein et al., 2008; Gajria et al., 2012; Johnston et al., 2009; Jagiello-Gruszfeld et al., 2010; Rugo et al., 2012).

The efficacy of lapatinib was evaluated in phase III trials, which led to its US Food and Drug Administration (FDA) approval in combination with capecitabine and in combination with letrozole for HER2/*neu*-positive MBC (Blackwell et al., 2010; Cameron et al., 2008; Di Leo et al., 2008).

Lapatinib, as a single agent or in combination with capecitabine, was also assessed for the treatment of brain metastases in patients with HER2/*neu*-positive MBC. Lapatinib alone resulted in objective CNS responses of 3% to 6%, while the addition of capecitabine resulted in an objective CNS response of 20% in patients who received prior whole-brain radiation (Lin et al., 2008). Several other studies of lapatinib plus capecitabine reported response rates of 31.8% to 38.5% (Lin et al., 2011; Sutherland et al., 2010). The combination of lapatinib plus capecitabine in patients with HER2/*neu*-positive MBC who have not received whole-brain radiation resulted in an objective response of 57% (Bachelot et al., 2013).

**Neratinib** 

Neratinib, a highly selective irreversible inhibitor of the kinase activity of HER2/*neu* and EGFR, showed antitumor activity as a single agent in patients with trastuzumab-pretreated MBC (Burstein et al., 2010; Tsou et al., 2005). Phase I/II trials evaluating the safety and efficacy of neratinib plus vinorelbine or paclitaxel in HER2/*neu*-positive MBC patients previously treated with anti-HER2/*neu* therapy reported the maximum tolerated dose (MTD) of neratinib to be 240 mg with promising antitumor activity, with an OR rate of 57% and 71%, respectively, and no unexpected toxicities (Chow et al., 2010; Awada et al., 2013).

Currently, neratinib is being studied in combination with temsirolimus (Torisel) in HER2/*neu*-positive or triple- negative MBC, as monotherapy vs. lapatinib plus capecitabine in trastuzumab pretreated HER2/*neu*-positive MBC, and in combination with paclitaxel vs. paclitaxel plus trastuzumab in the first-line treatment of HER2/*neu*-positive MBC (ClinicalTrials.gov identifiers NCT01111825, NCT00777101, and NCT00915018, respectively). Neratinib is also being investigated in the adjuvant setting upon completion of trastuzumab-based therapy, as well as for neoadjuvant treatment in locally advanced HER2/*neu*-positive breast cancer (NCT01008150).

**Afatinib** 

Afatinib is an irreversible dual inhibitor of EGFR/HER1 and HER2 TKI (Minkovsky & Berezov, 2008). This first phase I study evaluated the feasibility of oral dosing of afatinib in patients with solid tumors for 14 days on followed by 14 days off treatment and determined the MTD to be 70mg once daily (Eskens et al., 2008). Another phase I trial assessing continuous administration of afatinib in patients with solid tumors recommended a phase II study dose of 50 mg once daily (Yap et al., 2010).

A phase II trial assessing the efficacy and safety of afatinib in extensively pretreated patients with HER2/*neu*- negative MBC found that afatinib had limited activity in HER2/*neu*-negative breast cancer (Schuler et al., 2012). Another phase II trial evaluated afatinib monotherapy in patients with HER2/*neu*-positive MBC after failure of trastuzumab treatment. Data demonstrated 11% of evaluable patients had a partial response, 37% had stable disease as best response, and 46% achieved clinical benefit. Median PFS was 15.1 weeks and median OS was 61.0 weeks. The data revealed that afatinib monotherapy has promising clinical activity in extensively pretreated HER2/*neu*-positive breast cancer patients whose disease had progressed following trastuzumab treatment (Lin et al., 2012).

Other ongoing phase II studies are currently assessing the activity of afatinib in HER2/*neu*-positive MBC alone or in combination with a variety of agents, such as vinorelbine, trastuzumab, lapatinib, and letrozole (ClinicalTrials.gov Identifiers NCT01325428 and NCT01531764, NCT01325428, NCT00826267, NCT00708214, respectively), in different settings, including brain metastases and as neoadjuvant therapy (NCT01441596 and NCT01594177, respectively). An ongoing phase III study is comparing the addition of afatinib or trastuzumab to vinorelbine in HER2/*neu*-positive MBC patients who progressed on or after one prior trastuzumab-based treatment regimen (NCT01125566).

**Side Effects** 

The most commonly observed side effects with all HER2/*neu* and EGFR inhibitors are acne-like rash or folliculitis, diarrhea, and fatigue. Palmar-plantar erythrodysesthesia, nausea, vomiting, and fatigue were frequently seen with lapatinib. Although not common, decreased left ventricular ejection fraction, prolonged QT interval, and hepatotoxicity have been reported. Patients receiving neratinib experienced more diarrhea, nausea, and vomiting, with diarrhea being the most frequent grade 3/4 adverse event (Blackwell et al., 2012; Awada et al., 2013; Bachelot et al., 2013; Blackwell et al., 2010; Burstein et al., 2008; Burstein et al, 2010.; Cameron et al., 2008; Chow et al., 2010; Chu et al., 2007; Di Leo et al., 2008; Eskens et al., 2008; Gajria et al., 2012; Jagiello-Gruszfeld et al., 2010; Johnston et al., 2009; Lin et al., 2008; Lin et al., 2009; Lin et al., 2011; Lin et al., 2012; Rugo et al., 2012; Schuler et al., 2012; Sutherland et al., 2010; Tsou et al., 2005; Yap et al., 2010). A list of side effects is found in Table 2.

**Table 2:**
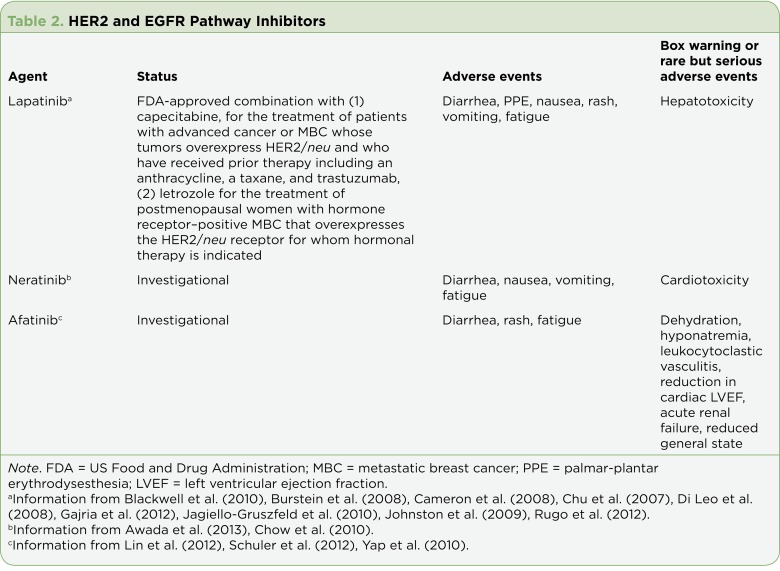
HER2 and EGFR Pathway Inhibitors

## Antiangiogenic Therapy

**Bevacizumab** 

Bevacizumab (Avastin) is a monoclonal anti-VEGF antibody that initially was approved for use with first-line paclitaxel in MBC. Its approval was based on a phase III trial (E2100) in which women with MBC received paclitaxel plus bevacizumab or paclitaxel alone, regardless of hormone receptor or HER2/*neu* status (Miller et al., 2007). The combination of bevacizumab and paclitaxel significantly increased PFS compared with paclitaxel alone (11.8 vs. 5.9 months, hazard ratio, 0.6; *p* < .001), and increased OS by investigator analysis (*p* < .01).

Several other phase III trials (AVF2119g, AVADO, RiBBOn-1, RiBBOn-2) demonstrated that bevacizumab added to chemotherapy significantly improved PFS, but not OS, compared with chemotherapy alone (Miller et al., 2007; Miller et al., 2005; Miles et al., 2010; Brufsky et al., 2011; Robert et al., 2011). A meta-analysis of all phase III studies confirmed the lack of survival benefit combined with the potential for serious adverse events and led the FDA to withdraw approval of bevacizumab for this setting in 2012 (Valachis et al., 2010).

**Side Effects** 

The most common adverse reactions observed in patients receiving bevacizumab at a rate > 10% are epistaxis, headache, hypertension, rhinitis, proteinuria, taste alteration, dry skin, rectal hemorrhage, lacrimation disorder, back pain, and exfoliative dermatitis. In clinical trials evaluating the efficacy and safety of bevacizumab in patients with breast cancer, cerebrovascular ischemia was the most significant grade 3 adverse event, together with proteinuria (2%), arterial throboembolitic events (0.8%), neuropathy (23.6%), infection (9.3%), fatigue (8.5%), and headache (2.2%; Miles et al., 2010; Miller et al., 2007; Miller et al., 2005; Brufsky et al., 2011; Robert et al., 2011). Other side effects less likely to occur are listed in Table 3.

**Table 3:**
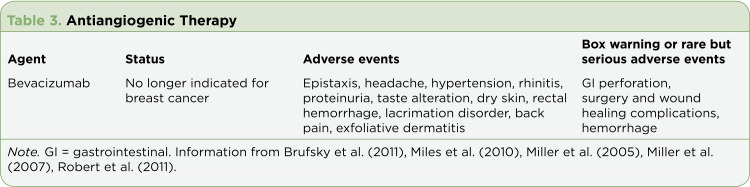
Antiangiogenic Therapy

## Poly(ADP Ribose) Polymerase Inhibitors

Poly(ADP ribose) polymerase (PARP) inhibitors, multifunctional enzymes involved in the mechanism of single- stranded DNA, stimulate early phases of DNA replication fork repair (Bryant et al., 2005). PARP inhibitors have selective anticancer activity in BRCA1- and BRCA2-deficient cancers (Farmer et al., 2005; Bryant et al., 2005). Several novel PARP inhibitors have proven to be beneficial in preclinical studies and are currently being investigated, such as BSI-201, AG014699, and ABT-888 (Gartner, Burger, & Lorusso, 2010).

**Side Effects** 

Most patients receiving PARP inhibitors experience mainly mild (grade 1/2) fatigue, nausea, vomiting, thrombocytopenia, and anemia. There were few patients who experienced grade 3 or higher toxicities including fatigue, nausea, thrombocytopenia, and anemia, but none required discontinuation of the drug (Plummer et al., 2005; Kummar et al., 2011). A list of side effects can be found in Table 4.

**Table 4:**
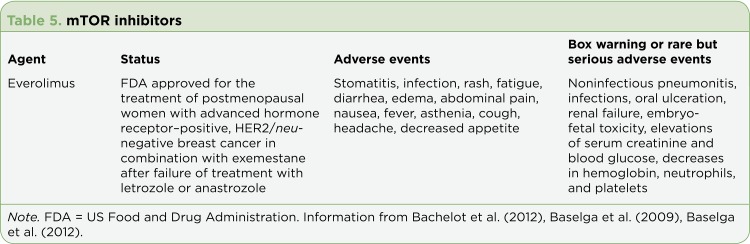
Poly(ADP Ribose) Polymerase (PARP) Inhibitors

## mTOR Inhibitors

**Everolimus** 

Everolimus (Afinitor) is an orally administered rapamycin derivative that inhibits the mTOR, a component of the signaling pathway that regulates cell growth and proliferation, metabolism, and angiogenesis (LoPiccolo, Blumenthal, Bernstein, & Dennis, 2008). The MTD of everolimus is 10 mg daily, as determined in a phase I study of everolimus in combination with carboplatin in MBC (Schwarzlose-Schwarck et al. 2012).

A randomized, double-blind phase II trial of postmenopausal women with operable ER-positive breast cancer receiving neoadjuvant treatment with letrozole and either everolimus (10 mg/day) or placebo revealed that everolimus increased response rate when compared to placebo (68% vs. 59%, *p* = .062; Baselga et al., 2009). The TAMRAD trial is a phase II trial that compared tamoxifen plus everolimus with tamoxifen alone in patients with hormone-receptor–positive, HER2-negative MBC with prior exposure to aromatase inhibitors (Bachelot et al., 2012). The clinical benefit rate, TTP, and OS were improved in the combination group (*p* = .045, *p* = .0021, and *p* = .007, respectively).

The randomized phase III trial BOLERO-2, which looked at everolimus plus exemestane vs. exemestane alone in patients with ER-positive, HER2/*neu*-negative MBC, found a significantly better PFS (*p* < .0001) and disease-free survival (*p* < .0001) in the combination group (Baselga et al., 2012).

Everolimus is currently under investigation in combination with lapatinib, vinorelbine, erlotinib, nab-paclitaxel, trastuzumab, cisplatin, paclitaxel, and letrozole in patients with MBC (ClinicalTrials.gov Identifiers NCT01272141, NCT01520103, NCT00574366, NCT00934895, NCT00912340, NCT00912340, and NCT01499160, respectively).

**Side Effects** 

The main side effects associated with the use of everolimus are stomatitis, pneumonitis, and metabolic abnormalities. The incidence of these side effects was even higher in combination therapy than with antihormonal therapy alone. In the phase III trial, a high percentage of patients discontinued everolimus due to a lack of tolerability (Baselga et al., 2012; Baselga et al., 2009; Bachelot et al., 2012). A list of everolimus side effects is available in Table 5.

**Table 5:**
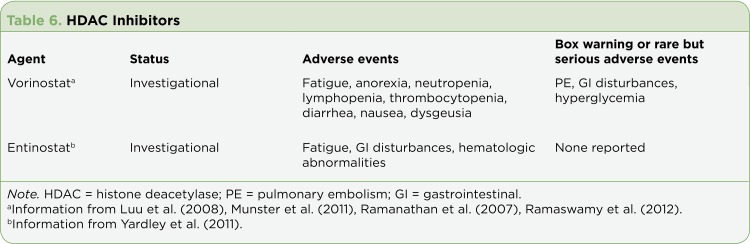
mTOR inhibitors

## HDAC Inhibitors

**Vorinostat** 

Vorinostat (Zolinza) is a small molecule that inhibits HDAC activity, stops proliferation, and induces differentiation and apoptosis (Almenara, Rosato, & Grant, 2002). The MTD of vorinostat was determined to be 400 mg or a twice-daily dose of 200 mg as monotherapy (Kelly et al., 2005; Kelly et al., 2003). Although vorinostat did not show antitumor activity as monotherapy, in phase II trials, encouraging anticancer activity was noted when it was combined with carboplatin and paclitaxel (Luu et al., 2008). The combination of vorinostat and tamoxifen was found to exhibit activity in reversing hormone resistance, with an OR rate of 19% and a clinical benefit rate of 40% (Munster et al., 2011). Vorinostat was evaluated for safety and efficacy in combination with paclitaxel and bevacizumab as first-line therapy in MBC. The overall response rate of 55% was similar to bevacizumab and paclitaxel alone, median PFS was 11.9 months, and median OS was 29.4 months in patients receiving vorinostat (Ramaswamy et al., 2012).

**Entinostat** 

Entinostat is a novel oral class I selective HDAC inhibitor that has been shown to inhibit breast cancer tumor growth, angiogenesis, and metastasis (Srivastava, Kurzrock, & Shankar, 2010). In ENCORE 301, a phase II study, the investigators evaluated the impact of the addition of entinostat to exemestane therapy on PFS in postmenopausal women with ER-positive MBC whose disease had progressed on a nonsteroidal aromatase inhibitor (Yardley, Ismail- Khan, & Klein, 2011). There was a significant improvement in PFS in the entinostat arm compared with placebo (4.28 vs. 2.27 months, respectively). In addition, preliminary results suggested that OS was significantly longer in the entinostat arm vs. the placebo arm (26.94 vs. 20.33 months, respectively). A phase III study is under way, as well as several phase II trials evaluating combinations of entinostat and azacitidine (NCT01349959), lapatinib (NCT01434303), and anastrozole or tamoxifen (NCT01234532) in women with metastatic or early-stage breast cancer.

**Side Effects** 

HDAC inhibitors are well tolerated, with primary toxicities including nausea/vomiting, fatigue, and a transient decrease in platelet and white blood cell counts. These effects are primarily mild (grade 1 or 2), transient, or reversible. Flattening or inversion of the T wave and QT prolongation have been observed in some patients; however, it is not known whether these effects have clinical relevance (Luu et al., 2008; Kelly et al., 2005; Kelly et al., 2003; Munster et al., 2011; Ramaswamy et al., 2012; Yardley et al., 2011). A thorough list of side effects can be found in Table 6.

**Table 6:**
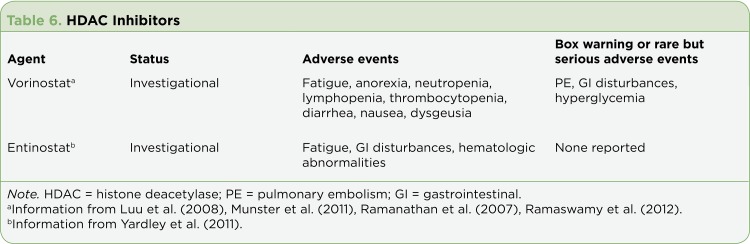
HDAC Inhibitors

## Heat Shock Protein

**Tanespimycin** 

Tanespimycin is a potent and selective heat shock protein 90 (Hsp90) chaperone inhibitor that causes degradation of client proteins. Tanespi- mycin plus trastuzumab was found to be well tolerated at a dose of 450 mg/m^2^ and to have antitumor activity in patients with solid tumors (Modi et al., 2007). In a phase II study, tanespimycin plus trastuzumab had significant anticancer activity in patients with HER2/*neu*-positive MBC previously progressing on trastuzumab (Modi et al., 2011).

**Side Effects** 

The most common side effects associated with tanespimycin are diarrhea, fatigue, nausea, headache, and neuropathy, yet these are predominantly mild (grades 1 and 2). There are few grade 3 drug-related side effects, such as diarrhea, fatigue, nausea/vomiting, headache, cough, and elevated liver enzymes, yet none resulted in the discontinuation of therapy in the phase II trial (Modi et al., 2007; Modi et al., 2011). A list of side effects can be found in Table 7.

**Table 7:**
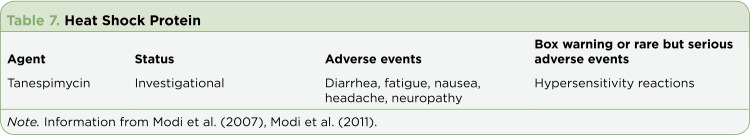
Heat Shock Protein

## Cyclin-Dependent Kinase Inhibitors

**Palbociclib** 

The orally available pyridopyrimidine-derived CDK-4 and CDK-6 inhibitor palbociclib (PD-0332991) has shown antineoplastic activity against breast cancer. A phase I/II study evaluating the safety and pharmacokinetics of palbociclib in postmenopausal women with ER-positive, HER2/*neu*-negative MBC determined the recommended phase II dose to be 125 mg daily on a 3 weeks on/1 week off schedule in combination with letrozole 2.5 mg daily (Slamon et al., 2010).

The subsequent phase II trial that compared palbociclib in combination with letrozole vs. letrozole alone in women with ER-positive, HER2/*neu*-negative MBC reported a preliminary statistically significant improvement in median PFS (26.2 vs. 7.5 months, respectively; *p* < .001), with a clinical benefit rate of 68% vs. 44%, respectively (Finn et al., 2012). The final results of this trial were reported at the American Association of Cancer Research annual meeting, with the PFS in women receiving the combination treatment being 20.2 vs. 10.2 months in women who received letrozole alone. Overall survival showed a trend in favor of the combined treatment, but it was not statistically significant (Finn et al., 2014). Phase III trials were slated to start in 2013.

**Side Effects** 

Neutropenia is the most common adverse event associated with palbociclib, with grade 3 neutropenia occurring in 12% of the patients receiving palbociclib alone, yet this was not cumulative in most patients when compared with cycle 1. None of the patients who had grade 3 neutropenia required granulocyte colony-stimulating factors, and none had febrile neutropenia. The most common nonhematologic adverse events are fatigue (34%), nausea (24%), and vomiting (19.5%; Slamon et al., 2010; Finn et al., 2012). A list of adverse events can be found in Table 8.

**Table 8:**
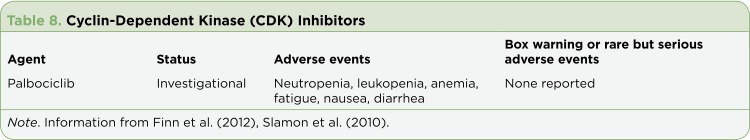
Cyclin-Dependent Kinase (CDK) Inhibitors

## Management of Side Effects

**Cardiac Toxicity** 

Cardiac toxicity is a concerning side effect of targeted therapy, especially anti-HER2/*neu* therapy. The majority of patients who develop cardiac dysfunction present solely with an asymptomatic reduction in left ventricular ejection fraction (LVEF). Generally, the cardiac dysfunction can be reversed with the discontinuation of treatment; however, a small percentage of patients experience progression to heart failure (Cobleigh et al., 1999). Patients with an asymptomatic decrease in LVEF should have their treatment discontinued and should be treated with angiotensin- converting enzyme (ACE) inhibitors or angiotensin II receptor blockers (ARBs) and beta-blockers. For patients with symptomatic heart failure, the addition of diuretics to the ACE inhibitors or ARBs and beta-blockers is recommended (Martín et al., 2009). Discontinuation of therapy is recommended in patients who develop clinically significant congestive heart failure (Martín et al., 2009). Thus, it is imperative that patients undergo a thorough baseline cardiac assessment prior to starting treatment, and that they are monitored frequently throughout the treatment.

**Diarrhea** 

A frequent side effect of targeted therapy is diarrhea, particularly when combined with chemotherapy. Diarrhea is also a dose-limiting toxicity (DLT) for most targeted therapies and can be a major cause of treatment discontinuation and decreased drug efficacy. Diarrhea can be easily managed with agents that decrease intestinal motility such as loperamide. Cancer treatment is rarely interrupted, yet reducing the dose of the drug may be necessary to lower the incidence and severity of the diarrhea (Widakowich, de Castro, de Azambuja, Dinh, & Awada, 2007; Wnorowski, de Souza, Chachoua, & Cohen, 2012). It is important to note that other causes of diarrhea, such as the use of laxatives, stool softeners, antacids or antibiotics, infection, partial intestinal obstruction or fecal impaction, should be excluded prior to treatment discontinuation or treatment dose reduction.

**Rash** 

A common skin toxicity of several targeted therapies, such as EGFR inhibitors, is an acneiform eruption. This side effect presents as erythematous follicular papules and pustules that appear predominantly on the face, upper chest, and back (Widakowich et al., 2007). Usually, the rash appears several days after the start of treatment and is more intense at weeks 2 or 3 of treatment. In most cases, the rash resolves after treatment discontinuation without sequelae. Withdrawing the treatment can be disquieting for patients, especially in light of data suggesting that the development of the rash is an indication of improved survival (Tang, Tsao, & Moore, 2006). Treatment of mild and moderate folliculitis includes hydration of the skin and use of topical and systemic antibiotics. Emollients, topical high-potency steroids, and topical immunomodulatory agents (tacrolimus and pimecrolimus) should only be used if xerosis and eczematous changes are present (Wnorowski et al., 2012).

## Adherence

Chemotherapy is generally administered intravenously by a trained nurse in an infusion area where patient adherence to treatment regimens can be readily assessed. In contrast, most targeted therapies are oral agents that are self-administered at home. Thus, the task of assessing patient adherence is more difficult in these cases. Recent studies have revealed that women with breast cancer have low adherence to tamoxifen (12%–59%), aromatase inhibitors (9%–50%), and chemotherapy (5%), respectively (Ruddy et al., 2012; Murphy et al., 2012).

Advanced practitioners can help patients get the right dose of their medications by laying out the dosing schedule in a clear manner, assuring that containers are well labeled, and providing regular telephone or text message reminders about taking their medications (Moore, 2007; Birner, Bedell, Avery, & Ernstoff, 2006). Educating patients and caregivers about the dosing schedule, the disease progression, what they should expect from the medications, the anticipated side effects, and how to manage them can also increase adherence (Jansen et al., 2007; Moore, 2007). In addition, it is imperative that advanced practitioners assess whether patients are taking their medications with each office or infusion visit.

## Conclusion

Treatment strategies for breast cancer have been steadily improving, in part due to the emerging investigation and understanding of the biological features of breast cancer. As advances are made in identifying targeted therapies, clinical practice has to undergo a transformation to accommodate the new side-effect profile. Advanced practitioners need to stay informed about the underlying biology and development of these novel cancer agents, educate patients about the agents and their side effects, and develop tools to assist patients with compliance. 
